# Phenothiazine-mediated rescue of cognition in tau transgenic mice requires neuroprotection and reduced soluble tau burden

**DOI:** 10.1186/1750-1326-5-45

**Published:** 2010-11-01

**Authors:** John C O'Leary, Qingyou Li, Paul Marinec, Laura J Blair, Erin E Congdon, Amelia G Johnson, Umesh K Jinwal, John Koren, Jeffrey R Jones, Clara Kraft, Melinda Peters, Jose F Abisambra, Karen E Duff, Edwin J Weeber, Jason E Gestwicki, Chad A Dickey

**Affiliations:** 1 Department of Molecular Medicine, Byrd Alzheimer's Research Institute, University of South Florida, Tampa, FL 33613, USA; 2 Department of Physiology and Pharmacology, Byrd Alzheimer's Research Institute, University of South Florida, Tampa, FL 33613, USA; 3 Department of Pathology, Life Sciences Institute, University of Michigan, Ann Arbor, MI, 48109, USA; 4 Department of Pathology, Taub Institute, Columbia University, New York, NY, 10032, USA

## Abstract

**Background:**

It has traditionally been thought that the pathological accumulation of tau in Alzheimer's disease and other tauopathies facilitates neurodegeneration, which in turn leads to cognitive impairment. However, recent evidence suggests that tau tangles are not the entity responsible for memory loss, rather it is an intermediate tau species that disrupts neuronal function. Thus, efforts to discover therapeutics for tauopathies emphasize soluble tau reductions as well as neuroprotection.

**Results:**

Here, we found that neuroprotection alone caused by methylene blue (MB), the parent compound of the anti-tau phenothiaziazine drug, Rember™, was insufficient to rescue cognition in a mouse model of the human tauopathy, progressive supranuclear palsy (PSP) and fronto-temporal dementia with parkinsonism linked to chromosome 17 (FTDP17): Only when levels of soluble tau protein were concomitantly reduced by a very high concentration of MB, was cognitive improvement observed. Thus, neurodegeneration can be decoupled from tau accumulation, but phenotypic improvement is only possible when soluble tau levels are also reduced.

**Conclusions:**

Neuroprotection alone is not sufficient to rescue tau-induced memory loss in a transgenic mouse model. Development of neuroprotective agents is an area of intense investigation in the tauopathy drug discovery field. This may ultimately be an unsuccessful approach if soluble toxic tau intermediates are not also reduced. Thus, MB and related compounds, despite their pleiotropic nature, may be the proverbial "magic bullet" because they not only are neuroprotective, but are also able to facilitate soluble tau clearance. Moreover, this shows that neuroprotection is possible without reducing tau levels. This indicates that there is a definitive molecular link between tau and cell death cascades that can be disrupted.

## Background

The current clinically available options for treating Alzheimer's disease (AD) are limited to acetylcholinesterase inhibitors and NMDA receptor antagonists [1,2]. For tauopathies like PSP and FTDP17, treatment is restricted to supportive therapies. Thus the demand to identify compounds that can remove the microtubule associated protein tau is extremely high.

Modifying tau patho-physiology has been the primary goal of first-generation tau therapeutics. For example, kinase inhibitors [3], microtubule stabilizers [4], tau aggregation inhibitors, immunotherapy [5], and chaperone-based drugs targeting disease-specific tau species [6], have all been proposed based largely on *in vitro *data. However, their efficacy for ameliorating cognitive deficits in mouse models of tauopathy have not been tested, primarily due to the fact that few models of tau accumulation are available that develop memory loss. One of the more controversial tau modifying compounds, to recently emerge as a potentially clinically relevant drug, is the phenothiazine methylthionium chloride better known as methylene blue (MB). MB is best known for its function in the laboratory as a redox indicator and as an antiseptic [7]; however, it, along with other phenothiazine derivatives, have been used extensively in the clinic since the 1950's to treat a number of different conditions, including schizophrenia, mania, anxiety, emesis, cancer, high blood pressure, allergies and even parasitic infections [8]. These compounds are generally well tolerated and have minimal side effects, including discoloration of urine and ocular vitreous.

More recently MB has been shown to inhibit the aggregation propensity of proteins that can adopt a β-sheet conformation *in vitro *[9-11], and it was this property that critically linked MB to AD as a possible plaque or tangle buster. However, the propensity of the phenothiazines to liberally bind to proteins and donate electrons has resulted in a number of other mechanisms being ascribed to them. For example, MB can regulate mitochondrial function [12,13] and inhibit Hsp70 ATPase activity [14,15].

Interestingly, these pleiotropic mechanisms and clinical applications combined with their relatively innocuous side effects and high bioavailability are what make the phenothiazines such an interesting therapeutic option for tauopathies. Most of the outcomes ascribed to MB could converge to ameliorate symptoms associated with tau accumulation. Here, we sought to determine whether MB could potentially be beneficial as a therapeutic option for tauopathies based on its pleiotropic anti-tau efficacy, we investigated how its chronic administration might impact the rTg4510 tau transgenic mice. We show that MB is capable of protecting neurons, however, only high dose MB treatment was able to reduce tau and also improve cognition. However, pathology was unaffected. This shows that MB does not reduce tau pathology, but reduces soluble tau levels. Also, it shows that neuroprotection alone is not sufficient to improve behavior, but only when MB levels are sufficiently high to reduce soluble tau levels can memory be improved.

## Results and Discussion

**Figure 1:**
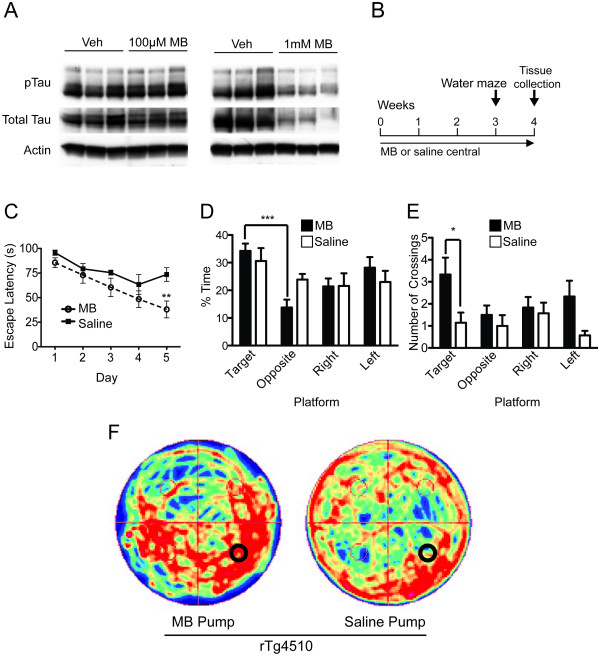
**Direct hippocampal infusion of methylene blue by mini-osmotic pump reverses spatial navigation related learning and memory deficits by reducing tau levels in rTg4510 mice**. (A) Four groups of wildtype mice (n = 3) were injected with 100 μM MB, 1 mM MB, or saline into the hippocampus for 24 hours. Western blot from hippocampal lysates show reductions in tau at 1 mM MB in phospho- and total tau. (B) Experimental design of central administration of MB (MB central n = 6, saline central n = 7). (C) Infusion of MB increased ability of rTg4510 mice to learn the location of the hidden platform in the MWM, **p < 0.01). (D) Probe trial of Morris water maze shows that MB treated rTg4510 mice were able to recognize the target from the opposite quadrant, unlike saline treated rTg4510 mice, (F(3,20) = 8.202, p = 0.0009), ***p < 0.001. (E) Number of crossing across the area where the hidden platform was located during training. (F(7, 44) = 2.757, p = 0.0183), p < 0.05. (F) Search strategy imaging using a camera tracking system shows that MB treated rTg4510 mice have a more focused strategy than saline treated rTg4510 mice.

**Figure 2:**
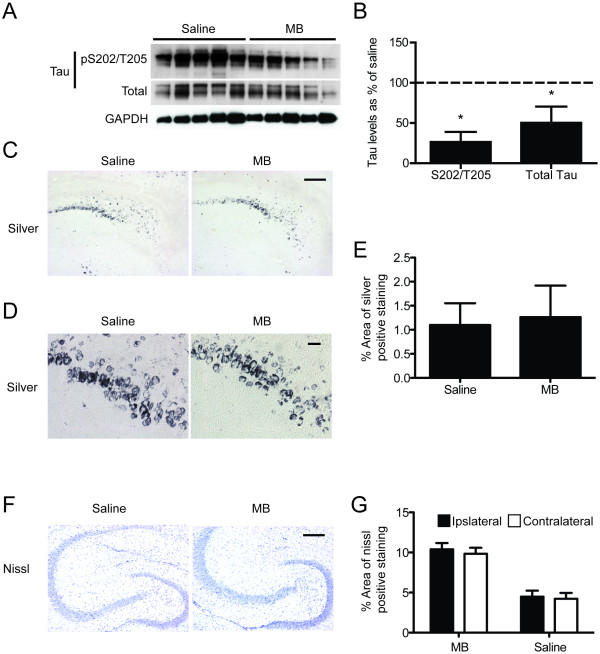
**MB treatment reduces tau levels, but does not affect pathology in mice with pre-existing tangle formation and neurodegeneration**. (A) Western blot analysis of tau protein levels from hippocampal lysates. (B) Quantitation of the optical density as a percentage of saline treated rTg4510 mice shows a significant difference in pS202/T205 and total tau levels, *p < 0.05. (C) Representative images of the gallyas silver stain. Scale bar, 200 μm. (D) High magnification images of the gallyas silver stain. Scale bar, 20 μm. (E) Percentage of area of silver positive staining shows no statistical difference between the means (n = 2 per group). (F) Representative images of the staining of neuronal nissl substance in the ipsilateral side of drug infusion (right hippocampus). Scale bar, 200 μm. (G) Quantitation of the percent area positive for nissl staining of ipsilateral versus the contralateral brain side of each group of mice shows that there is no detectable neuron loss due to pump implantation.

Previously, biochemical analyses of hippocampal tissue from tau transgenic mice (rTg4510; [16]) injected with MB showed reduced tau levels after 24 hours [15]. Hippocampi of wildtype mice were then injected with either 1 mM or 0.1 mM MB (based on a recent study in Zebrafish [17]) to determine its effective therapeutic window for reducing tau levels. Only 1 mM MB reduced tau levels (Figure 1A). Based on these results, 1 mM MB (1 mM; n = 6) or saline (n = 7) was administered to the right hippocampi [18] of 7-month old rTg4510 mice for 1 month using mini-osmotic pump implantation (Figure 1B). The accumulation of soluble mutant human tau in the forebrain of this model causes spatial memory deficits as early as 3 months of age, which precede neuronal loss [16]. Morris water maze (MWM) during the final week of treatment revealed that MB-treated mice showed significant improvements in learning the location of the escape platform compared to those receiving saline (Figure 1C). Probe trial analysis using target quadrant discrimination, number of platform crossings and search strategy imaging showed significantly improved cognitive recall in MB-treated mice compared to those treated with saline (Figure 1D-F). Biochemical analysis of hippocampal lysates showed that both phosphorylated (S202/T205) and total tau levels were significantly reduced (Figure 2A,B). Thus, despite the focal distribution of osmotic pump administration and the age of the mice, MB was still able to improve cognitive function; an effect that was concomitant with reductions in hippocampal tau levels. Contrastingly, pathology was unaffected (Figure 2C,D). Furthermore, nissl staining was done in order to determine if any damage was done to the hippocampus due to pump implantation. No such damage was found (Figure 2E,F).

**Figure 3:**
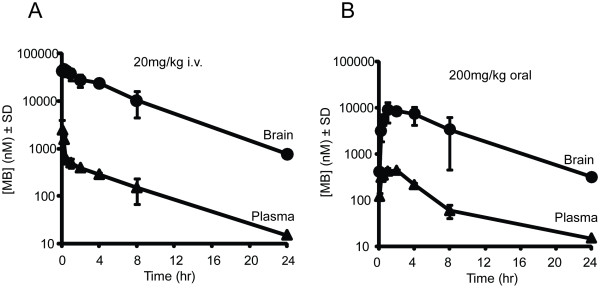
**Pharmacokinetic analysis of MB following peripheral administration**. (A) A single intravenous (I.V.) administration of 20 mg/kg MB was given to wildtype mice at different time points (n = 3 per time point), and cerebellar and plasma concentrations were measured through LC-MS. (B) A single 200 mg/kg oral gavage bolus of MB was given to wildtype mice at different time points (n = 3 per time point) and also measured by LC-MS. Cerebellar brain concentrations of MB are higher than plasma.

Based on this evidence, a new trial was initiated in rTg4510 mice to test the effects of long-term MB administration not only on behavior and tau biochemistry, but also on neuronal survival and tau pathology. Practical limitations with osmotic pump applications required that this study be done using non-invasive peripheral administration. Dose selection for this study was based on two factors: 1) FDA conversion tables show that a 10 mg/kg dose in a mouse is equivalent to ~1 mg/kg in humans, which is within the range of current MB clinical applications [19,20], and 2) pharmacokinetic analyses showed that MB could concentrate in the brain 500-fold, making the effective concentration. (> 100 μM) possible(Figure 3A,B)

**Figure 4:**
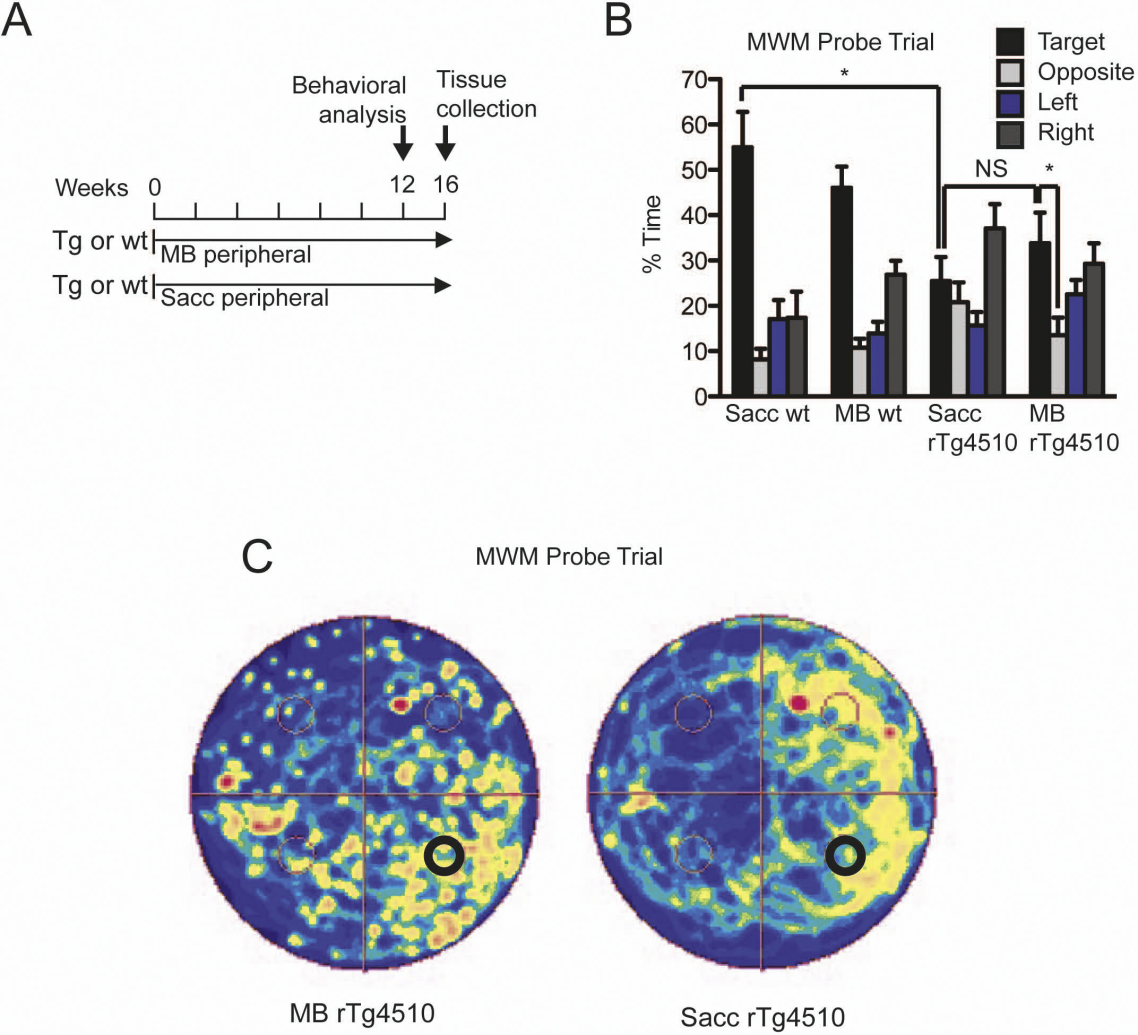
**Chronic treatment with methylene blue has moderate effect in behavior**. (A) Experimental design, n = 10. (B) Percent of time spent in each quadrant during the probe trial of MWM (F(15,144) = 8.781, p < 0.0001). MB rTg4510 recognize target versus opposite quadrant (F(3,36) = 3.38, p = 0.0286), *p < 0.05. (C) Search strategy imaging using a camera tracking system.

**Figure 5:**
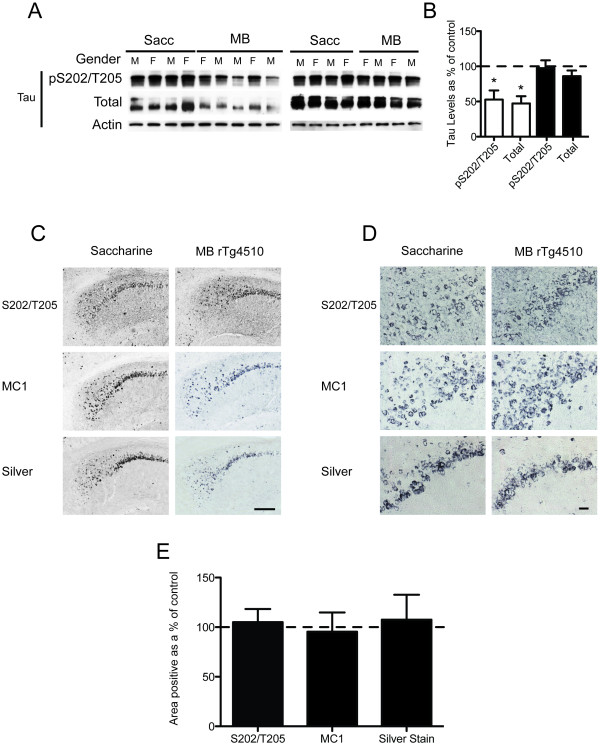
**Chronic dosing of methylene blue leads to reductions in soluble tau but pathology is unaffected**. (A) Half-brain lysates (no cerebellum) were analyzed for tau protein levels by Western blot. (B) Optical density of tau levels is shown as a percentage of control (saccharine treated rTg4510), *p < 0.05. (C) Representative images of immunoreactivity to a phosphorylated tau epitope (pS202/T205), the MC1 epitope (early tangles), and reactivity to the gallyas silver stain (late tangles) are shown. Scale bar, 200 μm. (D) High magnification images of immunohistochemistry. Scale bar, 20 μm. (E) Quantitation of the percent area of positive staining shows no statistical significance between the means of all stains.

Thus, two groups (n = 10) of 3 month-old rTg4510 mice and two groups of 10 wildtype littermates received either ~10 mg/kg(165 μM; 5× maximum recommended dose) of MB via drinking water supplemented with 2 mM saccharine or saccharine water alone (Figure 4A). Following 12 weeks of treatment, behavioral assessment showed no overt alterations in motor coordination or task acquisition (See additional file 1: Figure 1). MWM was then used to assess cognitive function. Probe trial analysis and search strategy imaging showed that MB, but not saccharine, prevented the significant progressive impairment in target quadrant discrimination that is a hallmark of the rTg4510 phenotype (Figure 4B,C). Furthermore, wildtype littermates showed normal spatial memory recall irrespective of MB treatment. Biochemical analyses of half-brain homogenates (excluding cerebellum) showed a reduction in soluble tau levels in some mice, but not others (Figure 5A,B). Histochemical analyses revealed no change in tau pathology in any mice (Figure 5C,D).

**Figure 6:**
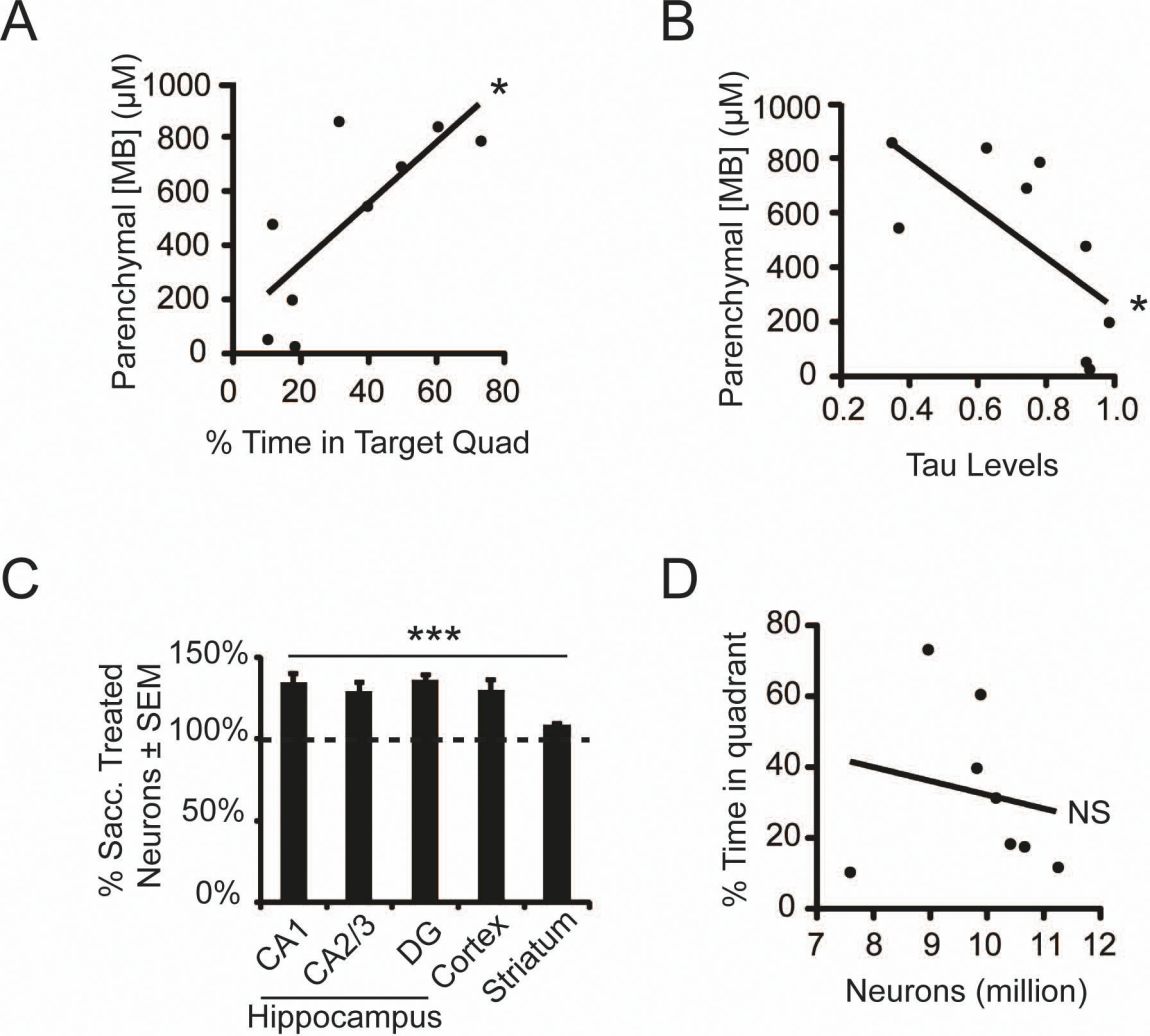
**Chronic dosing of methylene blue enhances neuronal survival**. (A) Parenchymal drug concentrations correlated significantly with memory retention in the probe trial of MWM p = 0.016, Pearson r = 0.766, r^2 ^= 0.587. (B) Tau levels inversely correlate significantly with parenchymal drug concentration, p < 0.05, Pearson r = -0.6724, r^2 ^= 0.452. (C) Number of neurons counted in five regions as a percentage of sacc treated rTg4510 (MB treated rTg4510 n = 8, sacc treated rTg4510 n = 10) (DG stands for dentate gyrus, CX stands for cortex and STR stands for striatum). (D) Number of neurons does not correlate significantly with time spent in the target quadrant.

Given the variability in behavioral performance as well as reductions in tau levels, we hypothesized that the MB concentration in the brain may have also varied due to the *ad libitum *administration strategy. To test this idea, brain concentrations of MB were assessed using LC-MS analysis of the cerebellar tissue from these mice. Indeed, MB concentration was positively correlated with MWM performance (p < 0.05) and was inversely correlated with soluble tau levels (p < 0.05) (Figure 6A,B). The differences in brain MB concentration between mice could not be attributed to body weight or gender (See additional file 1: Figure 2). Moreover, mice with >470 μM MB brain concentration accounted for a preponderance of the effects on memory function and tau reductions, consistent with our previous results showing that very high concentrations of MB were required for anti-tau efficacy (Figure 1A). Surprisingly, stereological assessment of five different brain regions from these mice showed that MB treatment significantly delayed neurodegeneration by ~30% in all forebrain regions of all rTg4510 mice (Figure 6C), but neuronal number failed to correlate with memory performance or soluble tau levels (Figure 6D and data not shown).

**Figure 7:**
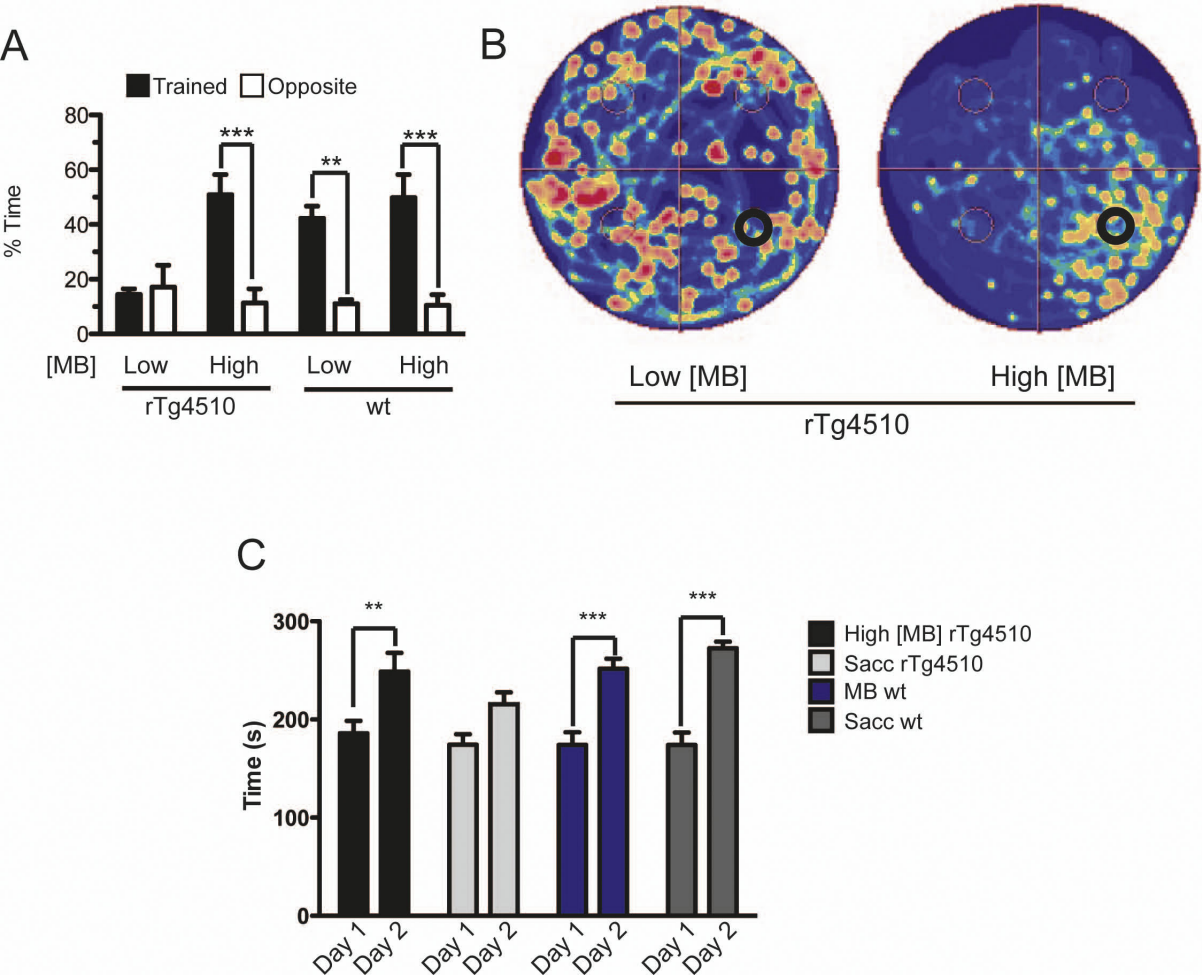
**High concentrations of MB repair spatial and motor memory deficits**. (A) (A)Post-hoc analyses reveal that High [MB] rTg4510 mice (n = 5) perform similar to wild-type MB treated mice, while Low [MB] rTg4510 mice (n = 4) did not display memory retention. Wildtype mice performed similarly regardless of MB (F(7,30) = 10.64, p < 0.0001), **p < 0.01, ***p < 0.001. (B) Camera tracking software imaging of MB treated rTg4510 mice divided by low and high parenchymal MB concentrations. (C) Motor learning was assessed by comparing the average latency to fall from the rotorod apparatus of day 1 with the average of day 2. High [MB] rTg4510 mice (n = 5) learn better than saccharine treated rTg4510 (n = 10). Wildtype groups, n = 10, **p < 0.01, ***p < 0.001.

Post-hoc analysis of the behavioral performances of rTg4510 mice with brain MB concentrations above or below ~470 μM (High [MB] or Low [MB], respectively), elucidated that the High [MB] cohort performed equivalent to wildtype mice, while the Low [MB] cohort was significantly impaired (Figure 7A). We again used the camera-tracking software to map the areas of the pool most traversed by each cohort (Figure 7B). High [MB] rTg4510 mice were predominantly found in the target quadrant, while the Low [MB] cohort displayed an unguided search strategy. Furthermore, we wanted to see if MB treatment in the High [MB] mice had an effect on motor learning. The mice were subjected to the rotorod task for two days and the latency to fall onto a spring-cushioned lever was measured. We found that motor learning from day 1 to day 2 of MB treated rTg4510 was significantly improved, dissimilar to the saccharine treated rTg4510 analogs (Figure 7C).

## Conclusions

In conclusion, these findings are consistent with recent evidence showing that reducing tangle burden does not beget functional recovery [16,21,22]; however, the unexpected result that neuroprotection is insufficient to preserve cognitive function suggests that preventing neuronal loss may not be enough to alter cognitive deficits in tauopathies either. In fact, it was only when soluble tau levels are reduced in the brain that functional recovery was observed. Moreover, these data show that there is a link between tau and cell death signaling cascades that can be altered with therapeutics since neurodegeneration can be decoupled from tau accumulation with MB.

## Materials and methods

### Mice

The rTg4510 mice and parental mutant tau and tTA lines were generated and maintained for this study as previously described [16].

### Central Administration

A concentration of 1 mM MB in saline was infused by pump into the CA3 of the right hippocampus of rTg4510 mice. Alzet pumps (Model 1004, 100 μL, 0.11 μl/hr; Alzet Osmostic Pumps) were filled with 100 μl of 1 mM MB or with saline 0.9%. The pumps were incubated in 0.9% saline at 37°C for 48 hrs. Mice were operated on a stereotaxic apparatus (51725 D, Stoelting, Wood Dale, IL). A midsagittal incision was made to expose the cranium and a small aperture was drilled with a dental tool over the right hippocampus to the following coordinates from bregma: anterior-posterior, -2.7 mm; lateral, -2.5 mm. The osmotic pump was inserted into a subcutaneous pocket on the back of the mouse, leading the catheter to the site of cannula (Brain infusion kit 3, Alzet) placement. The cannula, with a thin layer of cyanoacrylate (Loctite 454, Alzet), was attached to the stereotaxic cannula holder (Cannula holder 51636, Stoelting) and then lowered 3 mm ventral through the midline aperture. The incision was cleaned with saline and closed with surgical sutures. After surgery, the mice were housed individually. Infusion lasted for 28 days. Six mice were administered with MB and 7 with Saline. The mice were 7 months old at the time of surgery and they were 7 months and three weeks old at the time of water maze testing.

### Peripheral administration

Ten age and gender-matched rTg4510 mice and ten wildtype littermates were administered MB (Sigma) and saccharine (Acros Organics, Geel, Belgium) in their drinking water, while another ten rTg4510 mice and ten wildtype littermates received drinking water with saccharine only. A treatment of 650 mg/day in a 70 kg human equates to 9.3 mg/kg/day. Based on a 4.5 ml of water/day rate of consumption of a 30 g mouse, *ad libitum*, we estimated mice to receive 9.3 mg/kg/day (0.062 mg/ml). MB or vehicle administration was initiated in 12 week-old rTg4510 and wildtype mice using drinking water supplemented with 2 mM saccharine. Treatment was maintained for four months and the supplemented water was replaced three times per week. Mice were 6 months old at the time of initial behavioral assessment and they were 6.5 months old at the time of the water maze analysis. During the course of the study one mouse died of unknown circumstances and drug, tau levels and stereological analyses were not able to measured.

### Determination of Methylene Blue Concentrations in the Cerebellum

Frozen brain tissue was thawed on ice and homogenized for 2 minutes at a concentration of 100 mg/ml in homogenizing solution (ACN:PBS = 9:1). A 30 μL aliquot of the brain homogenate was added to a 1.5 mL polypropylene microcentrifuge tube and spiked with 30 μL of the internal standard solution (Methylene Violet 3RAX, 250 ng/ml). Analytical grade acetonitrile was then added (90 μL) and the sample was vortexed again for 30 s. Following highspeed centrifugation at 13,200 rpm for 10 minutes at 4°C, the supernatant was transferred to a glass vial and subjected to LC-MS analysis. The LC-MS system used for these studies was a Shimadzu (Columbia, MD) series 2010EV instrument equipped with an APCI probe to minimize ion suppression. Quantification was performed using LCMSolution Version 2.05 and a set of external standards.

### Behavioral Analysis

#### Open Field

The open field is used as a standard test of general activity. Animals are monitored for 15 minutes in a 40 cm square open field with a video tracking software (ANY-Maze, Stoelting, Illinois), under moderate lighting. General activity levels were evaluated by measurements of horizontal and vertical activity.

#### Rotorod test

This test was performed on an accelerating rotorod apparatus (Ugo Basile, Italy) with a 3 cm diameter rod starting at an initial rotation of 4 RPM accelerating to 40 RPM over 5 minutes. Mice were tested for the time spent on the rod during each of four trials with a thirty-minute inter-trial interval. Each trial was completed when the mouse fell off the rod (distance of 12 cm) onto a spring-cushioned lever.

#### Elevated Plus Maze

Anxiety can be assessed through the elevated plus maze (EPM). The EPM consisted of two well-lit open arms (35 cm) facing each other and two enclosed arms (30.5 cm) also facing each other. Each arm is attached to a common center platform (4.5 cm square) and elevated 40 cm off the floor. The mouse was placed in the center platform and allowed to explore for 5 min. Video tracking software measured movement in each section (ANY-Maze, Stoelting, Illinois).

#### Morris Water Maze

The Morris water maze (MWM) consisted of a circular pool (1.38-m diameter) filled with opaque water at room temperature with an escape platform (15 cm × 15 cm) hidden beneath the water level (3 cm). Each mouse was given four trials per day with an inter-trial interval of 1 hour for 6 consecutive days. The time to find the platform (escape latency), the total distance traveled and the swim speed of the animals was recorded. Each animal was given a maximum of 60 seconds to find the platform. During training, if the mice failed to find the platform after 60 seconds, they were placed on the platform for 30 seconds. They were then towel-dried and placed in a cage with a heating pad underneath until dry and returned to their home cage. On day 7 the mice were subject to one probe trial in which the platform was removed and each animal had 60 seconds to search the training pool for the platform.

### Brain Tissue Fractionation and Western Blot Analysis

Brain tissue was homogenized as previously described [23]. Measurements of tau levels were performed by western blot analysis.

### Immunohistochemistry

Fixed mouse brains were cryoprotected in successive 24 hours incubations of 10%, 20%, and 30% solutions of sucrose and then sectioned as previously described [Gordon, 2002]. Stained sections were imaged using an Olympus BX51 microscope at original 40×, 100×, or 200× final magnification. For quantification, images (original 100× magnification) of cornu ammonis (CA)1, CA3, entorhinal cortex, cortex and dentate gyrus were taken using spatial orientation cues. Quantification of positive staining product was determined using Image-Pro Plus (Media Cybernetics, Silver Springs, MD). Nissl staining was done in 0.05% cresyl violet for 5 minutes followed by differentiation in acidic water until desired color. Tissue was dehydrated through a graded series of ethanol (75%, 95% and 100%). Slides were cleared in Histoclear (xylene substitute) and coverslipped with DPX. Silver stain was performed as previously described [24].

### Stereological analysis

Neurons that were stained with cresyl violet were counted in the the cornu ammonis 1 (CA1), the cornu ammonis 2 and 3 (CA2+3), the dentate gyrus (DG), the cerebral cortex (CX) and the striatum (STR) using the optical fractionator method of stereological counting [25] with commercially available stereological software (StereoInvestigator, MBF Bioscience, Williston, VT). A systematic random sampling of sections throughout the left hemibrain were stained as described above and coded to ensure blinding. The regions of interests (ROI) were defined using specific landmarks within the brain to maintain consistency. A grid was placed randomly over the ROI slated for counting. At regularly predetermined positions of the grid, cells were counted within three-dimensional optical dissectors. Within each dissector, a 1 μm guard distance from the top and bottom of the section surface was excluded. Section thickness was measured regularly on all collected sections to estimate the mean section thickness for each animal after tissue processing and averaged 35.24 μm ± 0.46 μm for all sections analyzed. The total number of neurons was calculated using the equation:

$${\text{N = Q}}^{\text{-}}\times \text{1/ssf}\times \text{1/asf}\times \text{1/hsf}$$

where N is total neuron number, Q^- ^is the number of neurons counted, ssf is section sampling fraction, asf is the area sampling fraction and hsf is the height sampling fraction. Tissue from one mouse in the MB-treated rTg4510 cohort was unusable for this study. Therefore, n = 8 for this group.

### Antibodies

Tau antibodies S202/T205, MC1 and PHF-1 were provided by Dr. Peter Davies, Albert Einstein College of Medicine; total tau antibody was purchased from Stantacruz Biotech, Stantacruz, CA. Horseradish peroxidase conjugated secondary antibodies were obtained from Southern Biotech, Birmingham, AL. Glyceraldehyde-3-phosphate dehydrogenase antibody was obtained from Meridian Life Science, Saco, ME. Actin antibody was obtained from Sigma, St. Loius, MO. P-tau refers to PHF-1.

### Statistical Analysis

Statistical analysis comparing 2 groups was done using an unpaired, two tailed *t*-test. Analysis comparing more than 2 groups was done using a one-way analysis of variance (ANOVA) with the Tukey-Kramer post-hoc test. Analysis of three or more groups during different time points (water maze learning and rotorod) was done using repeated measures 2-way ANOVA with the Bonferroni post-hoc test. Statistical analysis was done in the GraphPad Prism software.

## Competing interests

The authors declare that they have no competing interests.

## Authors' contributions

CAD designed the study. JOL and CAD wrote the paper. JOL and CAD analyzed the data. JOL, QL, and LB executed most of the study. UJ, JK, AJ, JJ, CK, MP, and JA helped with aspects of the data collection. DW performed the stereology. PM performed the LC-MS. EC, KD, EW, and JG provided discussions on the results and commented on the manuscript. All authors read and approved the final manuscript.

## Supplementary Material

Additional file 1 **Supplementary data on the administration of methylene blue in rTg4510 mice**. Additional data contains information on the effect of chronic dosing of MB on behavior and statistical comparisons between parenchymal drug concentration, gender, and weight. Click here for file
