# Supplementary material for: Phenothiazine-mediated rescue of cognition in tau transgenic mice requires neuroprotection and reduced soluble tau burden
Source: Mol Neurodegener. 2010 Nov 1;5:45. doi: 10.1186/1750-1326-5-45 (PMC2989315; doi:10.1186/1750-1326-5-45)

Additional Figure 1. Chronic MB treatment does not cause abnormal changes in mice behavior.

(A) Mice were assessed in the open field task for abnormal locomotion and exploratory behavior for 30 minutes. No statistical differences were observed. (B) Mice were evaluated for anxiety in the elevated plus maze task. The amount of time spent in the open and closed arms, and the center area was evaluated and at least one mean was indicated to be significant (F (3,36) = 3.414, p<0.05). Post-hoc evaluation shows that MB treated wildtype mice displayed significantly more time in the closed arms and significantly less time in the open arms than both cohorts of transgenic mice. (C) Mice were subjected to the rotorod apparatus for 4 trials a day for 2 days. No overt differences were observed. (D) Mice positive for human P301L tau transgene expression and their wildtype littermates were grouped based on genotype and MB treatment. On the 14th week of chronic MB treatment they were subjected to the water maze paradigm. Learning was evaluated by the latency of the mice to find the hidden platform. The mice were trained until the wildtype mice plateaued. Wildtype animals learned significantly better than transgenic animals (n=10 per group, F (3, 236) = 19.71, p<0.0001). (E) Graphic representation of the mean latency of all mice in all trials of plot d.

Additional Figure 2. Differences in parenchymal drug concentrations could not be attributed to gender or weight.

(A) All drug treated mice (both transgenic and wildtype) separated by gender show no statistical difference between the means, p=0.7197 (males n=10, females n=9). (B) Linear regression and correlation between weight and drug concentration show that slope is not significantly non-zero and correlation is not statistically significant, p=0.7778 (n=19).

Additional Figure 1

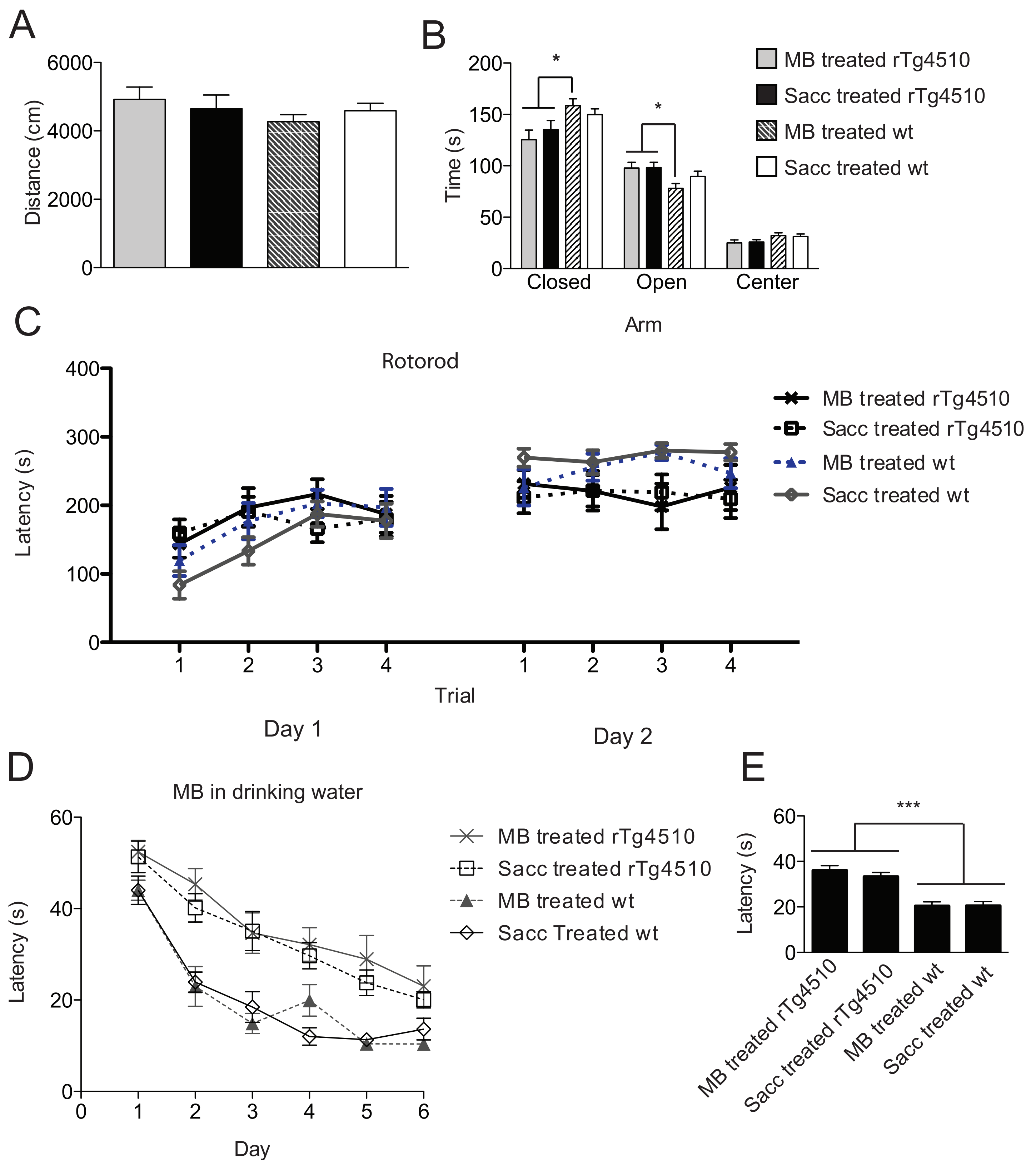

Additional Figure 2

A

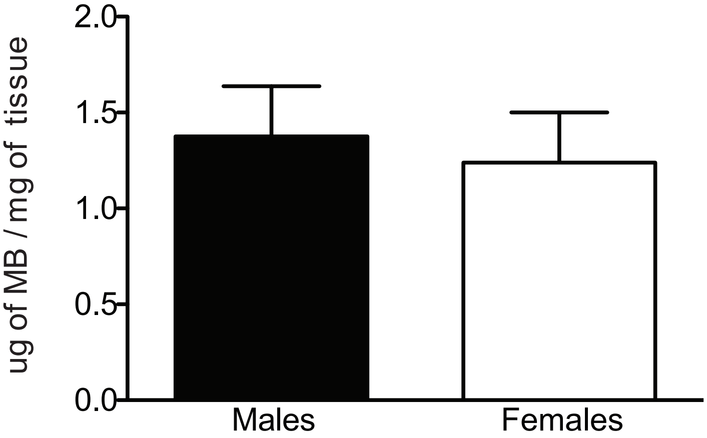

B

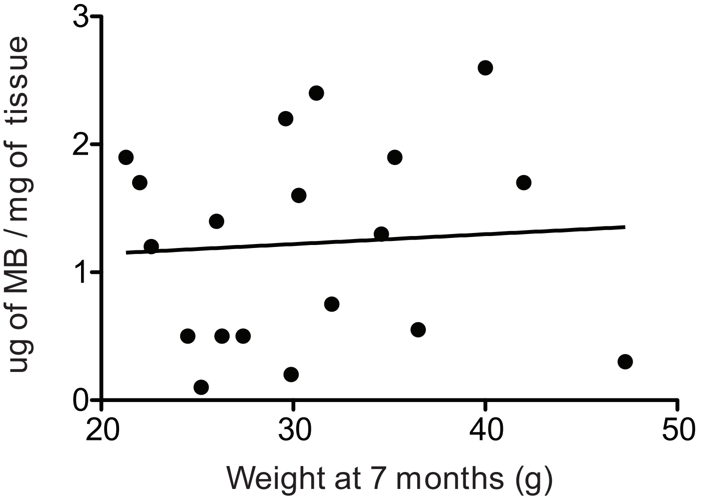

Supplement: Additional file 1 — Supplementary data on the administration of methylene blue in rTg4510 mice. Additional data contains information on the effect of chronic dosing of MB on behavior and statistical comparisons between parenchymal drug concentration, gender, and weight. [file 1750-1326-5-45-S1.PDF]
